# Evidence of progenitor cells in the adult human cochlea: sphere formation and identification of ABCG2

**DOI:** 10.6061/clinics/2017(11)11

**Published:** 2017-11

**Authors:** Milene Massucci-Bissoli, Karina Lezirovitz, Jeanne Oiticica, Ricardo Ferreira Bento

**Affiliations:** Departamento de Otorrinolaringologia, Faculdade de Medicina FMUSP, Universidade de Sao Paulo, Sao Paulo, SP, BR

**Keywords:** Progenitor Cells, Cochlea, ABCG2, Human

## Abstract

**OBJECTIVES:**

The aim of this study was to search for evidence of stem or progenitor cells in the adult human cochlea by testing for sphere formation capacity and the presence of the stem cell marker ABCG2.

**METHODS:**

Cochleas removed from patients undergoing vestibular schwannoma resection (n=2) and from brain-dead organ donors (n=4) were dissociated for either flow cytometry analysis for the stem cell marker ABCG2 or a sphere formation assay that is widely used to test the sphere-forming capacity of cells from mouse inner ear tissue.

**RESULTS:**

Spheres were identified after 2-5 days *in vitro*, and the stem cell marker ABCG2 was detected using flow cytometric analysis after cochlear dissociation.

**CONCLUSIONS:**

Evidence suggests that there may be progenitor cells in the adult human cochlea, although further studies are required.

## INTRODUCTION

Hearing loss is one of the most common sensorial deficiencies in humans. Although there exist several options for treating hearing loss, none of these approaches can completely reestablish auditory physiology. Prosthetic devices require maintenance and have inherent daily limitations due to their electrical nature; many such devices are simply not designed to be used while sleeping. One possible strategy to reestablish auditory physiology is the replacement of lost sensorial cells. In theory, the discovery of adult stem cells in the cochlea would bring this possibility closer to clinical practice.

No prior studies have attempted to find evidence of stem cells in the adult human cochlea. The availability of human cochlear tissue is limited, and most authors have chosen to use cochleas from patients who are undergoing surgical procedures that suggest permanent hearing loss 1-4. The only references to human cochlear stem cells involve fetal specimens 5-6.

Sphere formation assays have been used to demonstrate the proliferative capacity of neonatal mammalian cochleas in different species 7-9. Spheres have also been identified in adult mice, although such spheres were not numerous, could not differentiate *in vitro* and exhibited few developmental markers 10. In neonatal mice, there also exists evidence of a side population of cochlear cells that express the stem cell marker ABCG2 and are capable of *in vitro* proliferation, self-renovation and differentiation 11-14.

The aim of this study was to identify evidence of progenitor or stem cells in the adult human cochlea. To test our hypothesis, we used cochleas removed from patients undergoing vestibular schwannoma (VS) resection and brain-dead organ donors; this article is the first report involving the use of cochlear tissue from organ donors.

## MATERIALS AND METHODS

### Patients

Cochlear samples were obtained from patients undergoing excision of a VS via a translabyrinthine approach (n=2) and brain-dead organ donors (n=4) at University of Sao Paulo Clinics Hospital. All patients or their legal representatives provided informed consent prior to tissue collection in accordance with requirements of local and national ethics committees.

### Surgery in the VS group

A translabyrinthine approach was utilized by the same surgeon for both patients, and cochlear access was achieved as described by Browne and Fisch 15. After cochlear exposure, a wide cochleotomy was performed to allow for tissue removal. The membranous portion of the cochlea was collected into culture medium for immediate transport to the laboratory.

### Tissue collection in the organ donor group

To reach the cochlear promontory, three different approaches were tested. On one side, we used an endaural approach; on the remaining sides, retroauricular access was established to perform a mastoidectomy. On four sides, a posterior tympanotomy was conducted to gain access to the cochlear promontory. On one side, the posterior wall of the external auditory canal was drilled away to improve cochlear visualization. In all cases, after cochlear exposure, a wide cochleotomy was performed to allow for tissue removal. The membranous portion of the cochlea was collected into culture medium for immediate transport to the laboratory.

### Sphere formation assay

To test the ability of removed cochlear tissue to form spheres, we used the protocol described by Oshima et al. 16. Briefly, tissue was inspected with an inverted microscope, dissociated with trypsin, mechanically passed through a 70 µm filter and cultured under non-adherent conditions on defined media (DMEM-F12 with 2× B-27, 1× N2, 2 mM glutamine, 2 µL/mL ITS, 6 g/L glucose, 0.2 µL/mL ampicillin, 20 ng/mL EGF, 10 ng/mL bFGF and 50 ng/mL IGF) for up to five days.

### Flow cytometry

To verify the presence of the stem cell marker ABCG2, removed cochlear tissue was transferred to the laboratory in Eagle's minimum essential medium (EMEM; Vitrocell Embriolife, Campinas, SP, Brazil). Due to limited equipment availability, samples remained at 4°C for up to 36 hours; they were then dissociated with trypsin and mechanically dissociated 16 for antibody incubation in accordance with the manufacturer’s instructions.

We used an anti-ABCG2 antibody (Biolegend, 332020, San Diego, CA, USA) and its isotype (Biolegend, 401209), and flow cytometric analysis was performed on an Attune^®^ NxT Acoustic Focusing Cytometer (Thermo Fisher Scientific, Inc., Waltham, MA, USA).

## RESULTS

### Patients and tissue collection

The patients in the VS group (both female) were 61 and 63 years of age, and those in the organ donor group (2 males and 2 females) were 17-55 years of age. Both patients had moderate unilateral sensorineural hearing loss. In the organ donor group, six of the eight available sides were used. We have no auditory testing data from this group.

**Figure 1:**
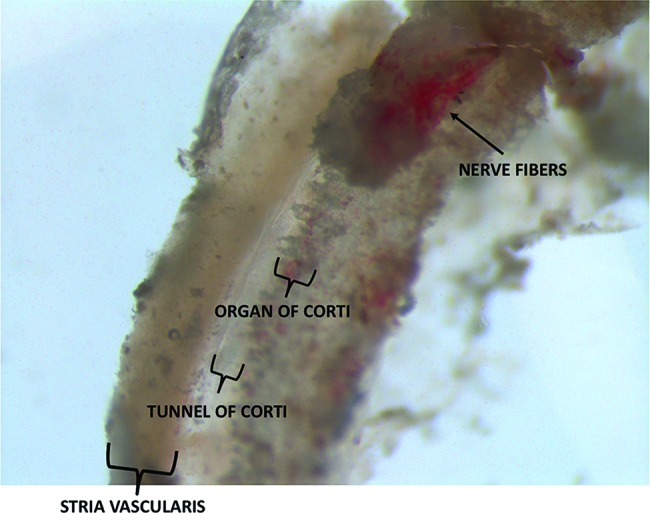
Membranous portion of the cochlea that was removed from a patient undergoing VS resection.

There was great variability in the quantities of tissue collected from patients and organ donors. In all cases, a portion of the organ of Corti and stria vascularis could be identified. In the organ donor group, the greatest quantity of tissue was collected when we used a retroauricular approach followed by mastoidectomy with drilling of the posterior wall of the external auditory canal. In the VS group, variability in tissue quantities was mainly attributable to anatomical variations. Figure 1 depicts a section of the membranous portion of the cochlea shortly after its removal from a patient undergoing VS resection.

### Identification of spheres

Three independent sphere formation assays were performed. Two of these assays used samples from the VS group (involving one cochlea each), and the remaining assay utilized a sample from the organ donor group (with two cochleas assessed in the same experiment). The evaluated cochleas were from 61- and 63-year-old female patients who were undergoing surgery and a 17-year-old male organ donor.

**Figure 2:**
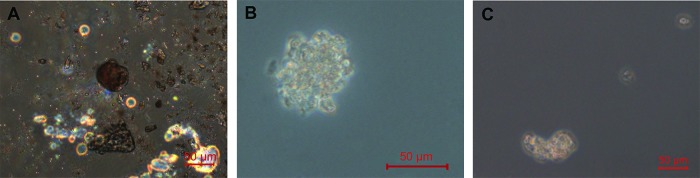
Sphere identification for samples from 2A, a 61-year-old female undergoing VS resection at 5 DIV; 2B, a 17-year-old male organ donor at 3 DIV; and 2C, a 17-year-old male organ donor at 5 DIV.

Spheres were identified in two of the three experiments (Figure 2).

### Identification of ABCG2

**Figure 3:**
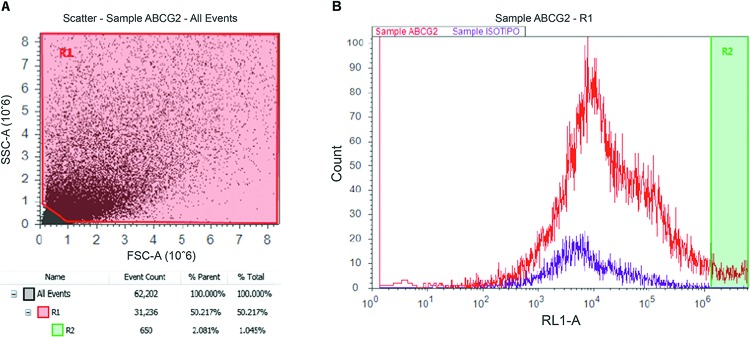
Flow cytometric analysis of a dissociated cochlea from a 33-year-old female (3B). There exists a population of cells positive for ABCG2 (R2) that represents 1.04% of the total cell population (3A).

Four cochleas from three organ donors were used for flow cytometric identification of ABCG2. The donors included one 38-year-old male and 33- and 55-year-old females. There was a clear population of ABCG2-positive cells in the dissociated cochlea from the 33-year-old female (Figure 3).

### Comparative table of results

 

## DISCUSSION

This article is the first report describing sphere formation and the identification of ABCG2 in the adult human cochlea and the first investigation in which brain-dead organ donors were a source of cochleas used for research.

The accurate identification of stem cells *in vivo* remains a major obstacle to understanding in stem cell biology since there are no single and universal stem cell markers common to all adult stem cells 17.

To identify evidence of stem or progenitor cells in the adult human cochlea, we used a sphere-forming assay validated for neonatal mice 16 that has been used to test the sphere-forming capacity of cells from adult mice 10. We identified spheres in two out of three experiments; nevertheless, the number of spheres was minimal, and no further testing could be adequately performed for further characterization.

Prior research has demonstrated that neonatal mice have a cochlear side population that expresses the stem cell marker ABCG2 11. Several authors have established that the direct identification of ABCG2 in this tissue is a marker of this side population 18-20. We have used flow cytometric analysis to reveal the presence of ABCG2 in adult human dissociated cochleas. We have been unable to sort and regrow ABCG2-positive populations due to the small number of cells obtained in our experiments, but testing to determine whether these cells behave similarly to mouse cochlear side population/ABCG2-positive cells would be extremely valuable 12-14.

A greater number of cochlear cells could be obtained for the aforementioned experiments via the collection of more cochleas from brain-dead organ donors, the standardization of the procedure for membranous cochlear resection, and the development of greater expertise with this procedure. Variability and little reproducibility in the removal of membranous cochlear tissue have been reported previously 1. However, with more widespread use of this material, this issue can be solved.

We have demonstrated evidence of the presence of progenitor cells in the adult human cochlea in the form of sphere formation and the detection of ABCG2. These findings do not conclusively establish the presence of stem cells in this tissue, but they do open a new field for stem cell research. We have reported the first use of brain-dead organ donors for cochlear resection for research purposes; this approach can be of inestimable value for hearing research.

## AUTHOR CONTRIBUTIONS

Bissoli MM was responsible for the manuscript preparation, cochlear preparation after its removal from patients and cell culture and staining for flow cytometry. Lezirovitz K was responsible for co-mentoring, manuscript preparation and figure selection. Oiticica J was responsible for the cochlear preparation after its removal from patients in selected cases and mentoring. Bento RF was responsible for the surgery, mentoring and manuscript preparation.

## Figures and Tables

**Table:** 

Age	Gender	Experiment	Result
61	female	sphere formation assay	negative
63	female	sphere formation assay	positive
17	male	sphere formation assay	positive
38	male	ABCG2 identification	inconclusive
55	female	ABCG2 identification	inconclusive
33	female	ABCG2 identification	positive
